# Evaluating fracture volume loss during production process by comparative analysis of initial and second flowback data

**DOI:** 10.1007/s40789-025-00754-9

**Published:** 2025-04-29

**Authors:** Chong Cao, Tamer Moussa, Hassan Dehghanpour

**Affiliations:** 1 Department of Civil and Environmental Engineering, University of Alberta, Edmonton, AB T6G 2W2 Canada; 2 Sinopec Petroleum Exploration and Production Research Institute, Beijing, 100083 China

**Keywords:** Second flowback data analysis, Infill development, Preloading effect, Effective fracture volume loss, Flowback rate-transient analysis

## Abstract

The fracture volume is gradually changed with the depletion of fracture pressure during the production process. However, there are few flowback models available so far that can estimate the fracture volume loss using pressure transient and rate transient data. The initial flowback involves producing back the fracturing fluid after hydraulic fracturing, while the second flowback involves producing back the preloading fluid injected into the parent wells before fracturing of child wells. The main objective of this research is to compare the initial and second flowback data to capture the changes in fracture volume after production and preload processes. Such a comparison is useful for evaluating well performance and optimizing fracturing operations.

We construct rate-normalized pressure (RNP) versus material balance time (MBT) diagnostic plots using both initial and second flowback data (FB_i_ and FB_s_, respectively) of six multi-fractured horizontal wells completed in Niobrara and Codell formations in DJ Basin. In general, the slope of RNP plot during the FB_s_ period is higher than that during the FB_i_ period, indicating a potential loss of fracture volume from the FB_i_ to the FB_s_ period. We estimate the changes in effective fracture volume (*V*_ef_) by analyzing the changes in the RNP slope and total compressibility between these two flowback periods. *V*_ef_ during FB_s_ is in general 3%–45% lower than that during FB_i_. We also compare the drive mechanisms for the two flowback periods by calculating the compaction-drive index (CDI), hydrocarbon-drive index (HDI), and water-drive index (WDI). The dominant drive mechanism during both flowback periods is CDI, but its contribution is reduced by 16% in the FB_s_ period. This drop is generally compensated by a relatively higher HDI during this period. The loss of effective fracture volume might be attributed to the pressure depletion in fractures, which occurs during the production period and can extend 800 days.

## Introduction

During the development and production of unconventional reservoirs, the fracture pressure gradually decreases as the fluid inside the fracture is gradually extracted. The changes of fracture characteristics (i.e., permeability, length, and width) with the depletion of fracture pressure are partially considered in the previous studies (Cao et al. 2022; Liu et al. 2018; Wang and Sharma 2019). Recent studies indicate that only about 25% of fracture length and 75% of effective fracture volume contribute to production over an extended production period (Gaddipati et al. 2020; Swami et al. 2017). The fracture length and permeability have been estimated by the production history matching (Jia et al. 2022; Lamidi Benson and Clarkson 2023). However, effective and robust methods for measuring fracture volume loss using flowback and production data are rarely reported.

Recently, flowback analysis has become a common practice for fracture characterization and well-performance evaluation. However, most studies have focused on characterizing the relationship between fracture permeability and pressure variation with the pressure depletion. The rate- and pressure-transient behaviors during flowback and post-flowback periods have been studied to understand the physics of flowback and to evaluate the fracture characteristics (Abbasi et al. 2014; Hossain et al. 2020, 2019; Ibrahim et al. 2019; Yang and Wu 2017). Flowback and early-time production analysis are increasingly used for fracture dynamics assessment, especially in shale gas development (Ibrahim et al. 2020; Zhang et al. 2023). Some pressure/rate transient models have been developed to investigate the effects of fracture half-length and fracture permeability on rate transient behavior (Clarkson and Williams-Kovacs 2013; Ezulike and Dehghanpour 2014; Gringarten 2008; Jia 2017; Li et al. 2019; Williams-Kovacs 2017; Zhang and Emami-Meybodi 2020a, 2020b). Moreover, several semi-analytical pressure-transient models have been applied for fracture volume estimation. The single- and multi-phase models for fracture volume estimation with pressure-supercharge effect have been proposed by Ezulike et al. 2016. Hossain et al. 2020 extended this tank model to analyze the effective stimulated reservoir volume using the post-flowback data. Moussa et al. 2020 estimated fracture volume with the changes of fracture porosity using the flowback data. Although many scholars have analyzed the changes of fracture permeability and geometry during the production processes, there remains a lack of quantitative multiphase flowback models to analyze the fracture volume loss caused by pressure depletion during production processes. Three phases (oil, gas, and water) may flow through the induced fractures, especially when the bottomhole pressure is lower than the bubble point pressure during flowback process. Therefore, three-phase flowback models are needed to be developed to capture the changes in fracture volume after long-term production processes.

Fracture closure, gas expansion, and water depletion have been considered as drive mechanisms for the flowback process. The results of Alkouh et al. 2014 show that the contribution of gas-drive mechanism (*S*_g_c_g_) is at least 97% when the water saturation is less than 70. Fracture compressibility is generally one to two orders of magnitude higher than matrix compressibility (Newman 1973; Sawabini et al. 1974). Consequently, fracture closure serves as a key drive mechanism during FB_i_ and FB_s_ periods. In this paper, we analyze various flowback-drive mechanisms to investigate the changes in fluid-flow mechanisms between FB_i_ and FB_s_ periods.

Although infill drilling has improved the production and economic return, these practices have led to growing concern over the side effect often referred to as fracture hits between parent and infill wells. The fracture hits can cause decreased production in a parent well, as well as other negative effects such as wellbore sanding and reduced production performance in the infill well (Ajisafe et al. 2021; Jacobs 2017; King et al. 2017; Whitfield et al. 2018). The preloading strategy has been proved to be an effective and successful way to mitigate the loss of production caused by fracture hit (Aniemena et al. 2022; Joslin et al. 2020; Whitfield et al. 2018). To limit the influence of parent–child fracture hit, water is usually injected into the parent wells before the stimulation of child wells. The preloading fluid is produced back to the surface (Whitfield et al. 2018; Zheng et al. 2021). This process is referred to as the second flowback (FB_s_). It provides sufficient hourly flowback data and daily production data to quantitatively assess the changes in fracture volume from the FB_i_ period to the FB_s_ period.

The objective of this paper is to capture the changes in effective fracture volume during the production periods between the two flowback processes and compare the rate and pressure transient responses of the second flowback period with those of the initial flowback period to evaluate the changes in fracture characteristics. First, the generalized multiphase (oil, gas, and water) flowback model for FB_s_ period is proposed to evaluate the fracture volume loss between the FB_i_ period and the FB_s_ periods. Then, the fracture volume loss is estimated from  the changes in the slope of RNP diagnostic plots and the variations of total compressibilities between FBi and FBs periods. Next, the RNP diagnostic plots of six multi-fractured horizontal wells completed in Niobrara and Codell formations in DJ Basin in the United States are constructed and the changes in fracture volume are compared. Finally, the compaction-drive index (CDI), hydrocarbon-drive index (HDI), and water-drive index (WDI) are used to compare the variations of production drive mechanisms during the two flowback periods.

## Workflow for fracture volume loss

### Timeline of initial and second flowback periods

**Fig. 1:**
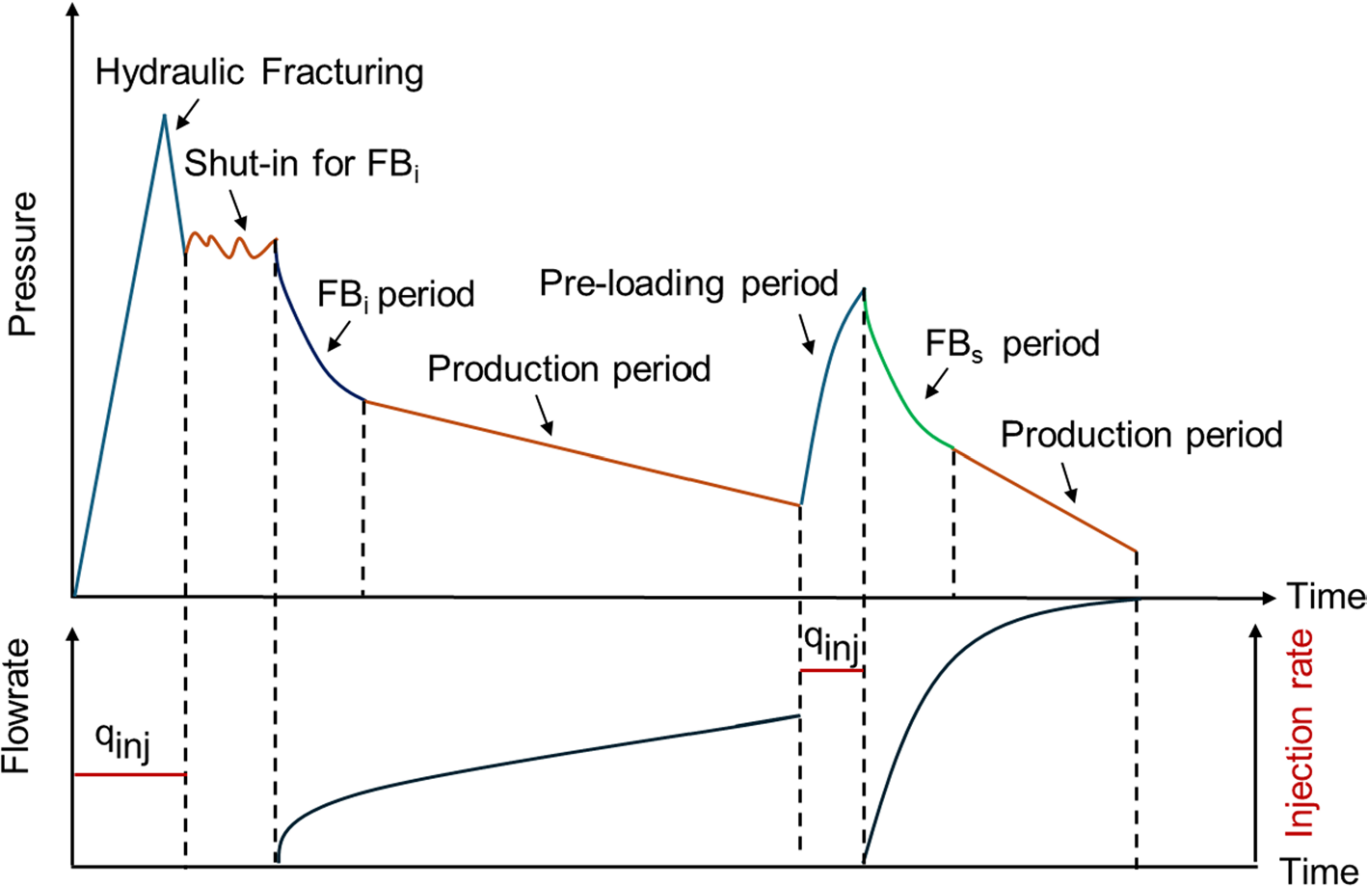
Schematic illustration of flow rate and pressure versus time profiles

Figure 1 illustrates the profiles of flow rate and pressure versus time from hydraulic fracturing treatment to the second flowback and subsequent production period. For the fractured horizontal wells in tight or shale reservoirs, the well is typically shut in for a period of time after hydraulic fracturing. This procedure allows more fracturing fluid to enter deeper into the reservoir and displace more oil. After the shut-in period, the well is then opened for the initial flowback (FB_i_), followed by the transition to later production. The fractures are generally filled with fracturing fluid at the beginning of the FB_i_ period. To mitigate pressure interference resulting from infill drilling, a large amount of water is injected into the parent well during the pre-loading period (Aniemena et al. 2022; Whitfield et al. 2018). In this work, the period during which the pre-loading fluid from the parent wells is produced back to the surface after the stimulation of child wells is referred to as the second flowback (FB_s_). The data from FB_i_ and FB_s_ periods are adopted in this paper to capture the loss of fracture volume due to pressure depletion during long-term production period.

### Fracture volume loss estimation

**Fig. 2:**
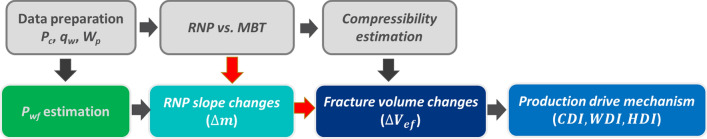
The workflow for the estimation of fracture volume changes

In this paper, we construct the rate-normalized pressure (RNP) diagnostic plots for both FB_i_ and FB_s_ periods. The average effective fracture volume can be estimated by the changes in RNP slope and total compressibility between FB_i_ and FB_s_ periods. The variations in effective fracture volume and production-drive mechanisms are compared for both FB_i_ and FB_s_ periods. The workflow for the estimation of fracture volume changes is shown in Fig. 2.

1. Flowback data preparations: We collect initial and second flowback data, including tubing and casing pressures, along with flow rate (*q*_w_) and cumulative water production (*W*_p_) of the parent wells. The hourly data from the initial flowback (FB_i_) and daily data from the second flowback (FB_s_) are then analyzed after noise removal.
2. Pressure estimations: The bottomhole pressure data are estimated from casing pressure by using the correlation of Hagedorn and Brown 1965. The initial average reservoir pressure is estimated by averaging the flattening value of the calculated downhole pressure before hydrocarbon breakthrough as proposed by Jones et al. 2014.
3. RNP diagnostic analysis: We construct the diagnostic plots of the RNP versus material-balance time (MBT) for both FB_i_ and FB_s_ data of the studied wells. RNP and MBT during FB_i_ and FB_s_ periods can be described as (Hossain et al. 2019)
  1 $$\text{RNP}_{{{\text{FB}}_{{\text{i}}} }} = (\frac{{p_{\text{i}} - p_{\text{wf}} }}{{q_{\text{w}} }})_{{_{{{\text{FB}}_{{\text{i}}} }} }} ,\text{RNP}_{{{\text{FB}}_{{\text{s}}} }} = (\frac{{p_{\text{i}} - p_{\text{wf}} }}{{q_{\text{w}} }})_{{_{{{\text{FB}}_{{\text{s}}} }} }}$$
  2 $$\text{MBT}_{{{\text{FB}}_{{\text{i}}} }} = (\frac{{W_{\text{p}} }}{{q_{\text{w}} }})_{{{\text{FB}}_{{\text{i}}} }} , \, \text{MBT}_{{{\text{FB}}_{{\text{s}}} }} = (\frac{{W_{\text{p}} }}{{q_{\text{w}} }})_{{_{{{\text{FB}}_{{\text{s}}} }} }}$$
  Here, the subscripts FB_i_ and FB_s_ represent the initial and second flowback periods, respectively.
4. Average effective fracture volume estimation: The RNP slope (*m*) for both FB_i_ and FB_s_ periods can be determined from the RNP versus MBT diagnostic plots. The detailed derivation of the slope of the rate-normalized pressure (RNP) curve is provided in the model section, and it is expressed as
  3 $$m({\text{slope}}) = \frac{1}{{V_{\text{ef}} c_{\text{t}} }}$$
  Based on Eq. 3, the average effective fracture volume (*V*_ef_) is determined by utilizing the RNP slope and the total compressibility(Fu et al. 2017). The total compressibility is a function of the fracture, water and oil compressibility and water saturation. We assume that the water compressibility does not change between the FB_i_ and FB_s_ periods. *c*_w_ is obtained from the existing correlations (Meehan 1980), and *c*_o_ is obtained from the pressure-dependent compressibility curves for the Niobrara Formation (Ning et al. 2018). The water saturation (*S*_wi_) of the FB_i_ period is assumed 1 since there is a long period of single-phase water production at the beginning of the FB_i_ period. *S*_w_ of the FB_s_ period can be estimated by (Ezulike and Dehghanpour 2014)
  4 $$S_{\text{w}} = S_{\text{wi}} - \frac{{Q_{\text{w}} }}{{V_{\text{ef}1} }}$$
  Here, *Q*_w_ is the water production volume from the FB_i_ period to the start of FBs period.
  The primary challenge in estimating *V*_ef_ ​ lies in accurately calculating the fracture compressibility. Here, we adopt the graphical method proposed by Aguilera 1999 to estimate *c*_pe,_ as shown in Appendix C. *c*_pe_ is estimated using net pressure and mineralization values. The net pressure (*p*_n_) can be calculated by (Sherman and Holditch 1991)
  5 $$p_{\text{n}} = p_{\text{closure}} - p_{\text{wf}}$$
  Here, *p*_closure_ is the fracture closure pressure that can be approximated by the minimum horizontal principal stress. The maximum pressure during the shut-in period is used to approximate the fracture closure pressure in the absence of diagnostic pressure injection test (DFIT) (Wang et al. 2019). Therefore, the average effective fracture volume is estimated using the RNP slope, *m*, water saturation, *S*_w_, average fracture compressibility,* c*_pe_, water compressibility, *c*_w_, and oil compressibility, *c*_o_.
5. Comparing flowback drive mechanisms: Based on the definitions of total compressibility, the drive mechanisms of fracture closure, hydrocarbon expansion and water depletion can be evaluated quantitatively. The drive mechanisms for the two flowback periods are compared by calculating three flowback-drive indices (compaction-drive index (CDI), hydrocarbon-drive index (HDI), and water-drive index (WDI)) introduced by Ezulike et al. 2016:
  6 $$\text{CDI} = \frac{{c_{\text{pe}} }}{{c_{\text{t}} }}$$
  7 $$\text{HDI} = \frac{{S_{\text{o}} c_{\text{o}} + S_{\text{g}} c_{\text{g}} }}{{c_{\text{t}} }}$$
  8 $$\text{WDI} = \frac{{S_{\text{w}} c_{\text{w}} }}{{c_{\text{t}} }}$$
  Here, the sum of HDI and WDI represents fluid expansion, which can be expressed as EDI = HDI + WDI.
6. Effective fracture volume and total compressibility changes: The loss percentage of effective fracture volume (*R*_Vef_) and total compressibility (*R*_ct_) during the two flowback periods can be estimated by
  9 $$R_{{V_{\text{ef}} }} = \frac{{V_{\text{ef}1} - V_{\text{ef}2} }}{{V_{\text{ef}1} }}$$
  10 $$R_{{c_{\text{t}} }} = \frac{{c_{\text{t}1} - c_{\text{t}2} }}{{c_{\text{t}1} }}$$
  Here, *V*_ef1_ and *V*_ef2_ are the effective fracture pore volume estimated by analyzing the initial and second flowback data, respectively. *c*_t1_ and *c*_t2_ are the total compressibility estimated by analyzing the initial and second flowback data, respectively.

## Methodology

### Conceptual model

**Fig. 3:**
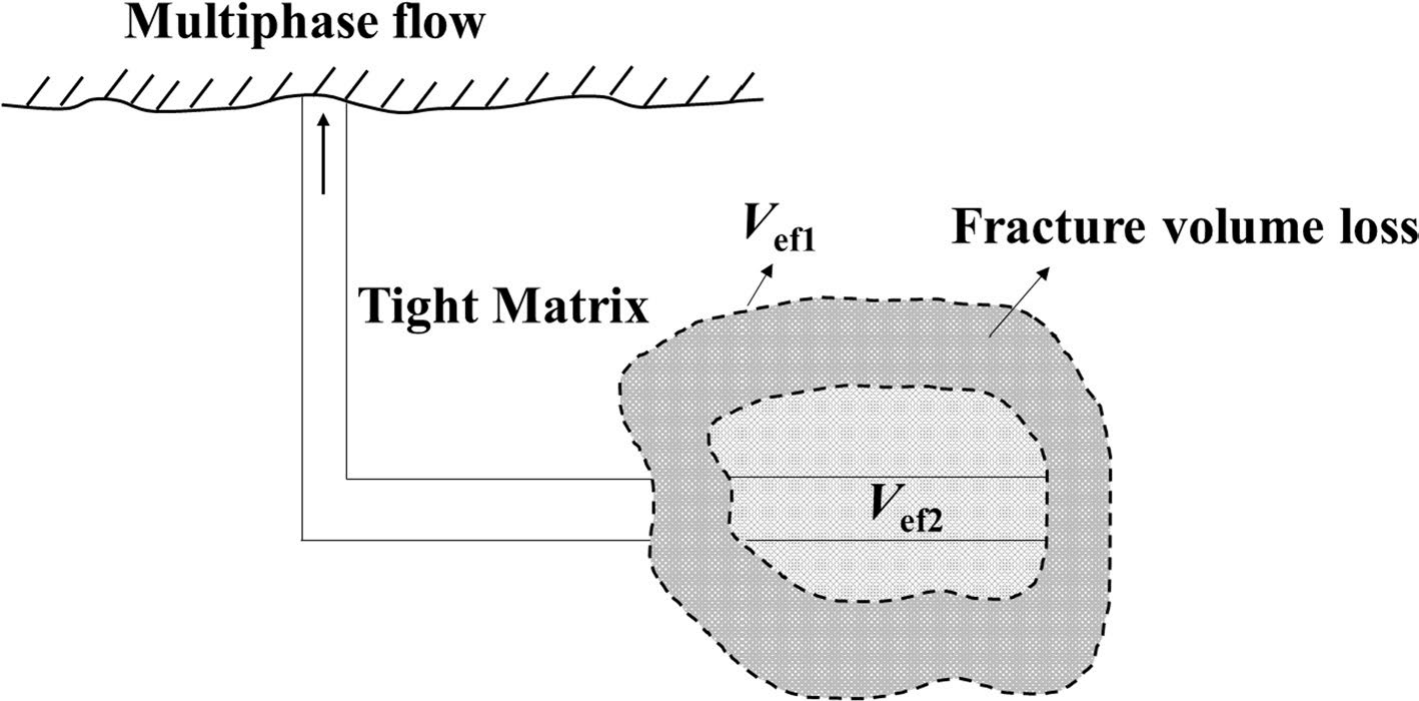
Schematic illustration of fracture volume loss between FBi and FBs periods

At the beginning of the FB_i_ period, the fractures are almost filled with fracturing water, and the fracture volume is denoted as *V*_ef1_. After a period of production, the pressure in the fractures gradually decreases. The fracture volume gradually shrinks from *V*_ef1_ to *V*_ef2_ between FBi and FBs period, as shown in Fig. 3. The FB_i_ data from the single-phase water production can be used to estimate the initial fracture volume. During the FBs stage, fluids (oil, gas and water) are gradually extracted, resulting in multiphase flow (oil, gas and water) within the fractures. Three-phase (oil, gas and water) flow occurs in the fractures especially when the bottomhole pressure drops below the bubble point pressure. The fracture volume after a long production period can be estimated from the FB_s_ data. Therefore, the fracture volume loss can be determined by comparing the fracture volume changes between FB_i_ and FB_s_ periods.

This paper hypothesizes that the changes in effective fracture volume can be evaluated by analyzing and comparing the initial and second flowback. Here, we assume that 1) the fractures are initially filled with water during FB_i_ period; 2) the fractures are filled with multiphase fluids (water + oil/gas) after injecting the preload fluids into the parent wells, and these multiphase fluids are primarily produced from the fractures during FB_s_ period; and 3) the gas expulsion from the oil is negligible under downhole conditions during the FB_i_ period. First, we test the hypothesis by analyzing the pressure and rate profiles measured during the two periods for six fractured horizontal wells. Then, we construct and compare RNP diagnostic plots for the initial and second flowback periods. Finally, we evaluate the changes in effective fracture volume by analyzing the variations in total compressibility and the slope of RNP versus MBT plots.

### Multiphase flowing material balance model for FB_s_ period

The gas expulsion from the oil under downhole conditions is considered for the second flowback period in this work. Therefore, a multiphase (oil, gas, and water) flowback model is developed in this paper. The water-phase mass conservation equations for both FB_i_ and FB_s_ in the fractures are described as (Hossain et al. 2020, 2019)

11 $$q_{{\text{w}_{{\text{in }}} }} \rho_{\text{w}} - q_{{\text{w}_{{\text{out }}} }} \rho_{\text{w}} = \frac{\partial }{\partial t}\left( {\rho_{\text{w}} V_{\text{w}} } \right)$$

Since the water influx from the matrix is negligible as assumed in the previous section, Eq. (11) can be rewritten as

12 $$0 - q_{\text{w}} \rho_{\text{w}} = \frac{\partial }{\partial t}\left( {\rho_{\text{w}} V_{\text{w}} } \right)$$

The water volume in the effective fracture volume is given by

13 $$V_{\text{w}} = V_{\text{ef}} - V_{\text{o}} - V_{\text{g}}$$

Equation (12) is rewritten using the definition of *V*_w_ as

14 $$- q_{\text{w}} \rho_{\text{w}} = V_{\text{w}} \frac{{\partial \rho_{\text{w}} }}{\partial t} + \rho_{\text{w}} \left( {\frac{{\partial V_{\text{ef}} }}{\partial t} - \frac{{\partial V_{\text{o}} }}{\partial t} - \frac{{\partial V_{\text{g}} }}{\partial t}} \right)$$

Equation (14) can be further expanded using the chain rule as

15 $$- q_{\text{w}} \rho_{\text{w}} = V_{\text{w}} \frac{{\partial \rho_{\text{w}} }}{\partial P} \times \frac{\partial P}{{\partial t}} + \rho_{\text{w}} \left( {\frac{{\partial V_{\text{ef}} }}{\partial P} \times \frac{\partial P}{{\partial t}} - \frac{{\partial V_{\text{o}} }}{\partial P} \times \frac{\partial P}{{\partial t}} - \frac{{\partial V_{\text{g}} }}{\partial P} \times \frac{\partial P}{{\partial t}}} \right)$$

Here, oil and gas compressibility, defined as the changes in volume with respect to pressure, is simplified as the volume variation with pressure. The oil and gas compressibility can be defined as

16 $$c_{\text{g}} = - \frac{1}{{V_{\text{g}} }}\frac{{\partial V_{\text{g}} }}{\partial P}$$

17 $$c_{\text{o}} = - \frac{1}{{V_{\text{o}} }}\frac{{\partial V_{\text{o}} }}{\partial P}$$

Based on the definition of fracture, water, oil, and gas compressibility in Appendix A, Eq. (15) can be written as

18 $$- q_{\text{w}} = V_{\text{w}} c_{\text{w}} \frac{\partial P}{{\partial t}} + \left( {V_{\text{ef}} c_{\text{f}} + V_{\text{o}} c_{\text{o}} + V_{\text{g}} c_{\text{g}} } \right)\frac{\partial P}{{\partial t}}$$

The equation is further expanded as

19 $$- q_{\text{w}} = V_{\text{ef}} \left[ {c_{\text{f}} + S_{\text{w}} c_{\text{w}} + S_{\text{o}} c_{\text{o}} + S_{\text{g}} c_{\text{g}} } \right]\frac{\partial P}{{\partial t}}$$

Based on the definition of total compressibility, Eq. (19) can be written as

20 $$- q_{\text{w}} = V_{\text{ef}} c_{\text{t}} \frac{\partial P}{{\partial t}}$$

Integrating both sides of Eq. (20), we have

21 $$\text{RNP}_{\text{w}} = \frac{1}{{V_{\text{ef}} c_{\text{t}} }}\text{MBT}_{\text{w}}$$

The corresponding mathematical models and detailed derivations are provided in Appendix A. From Eq. (21), the changes in RNP slope represent the combined changes in both *V*_ef_ and *c*_t_.

Here, RNP_w_ is the difference between the initial reservoir pressure (*p*_i_) and the flowing bottomhole pressure (*p*_wf_) divided by the water rate (*q*_w_). MBT_w_ denotes the sum of cumulative water production (*W*_p_) divided by the water rates. RNP_w_ and MBT_w_ are respectively given by

22 $$\text{RNP}_{\text{w}} = \frac{{p_{\text{i}} - p_{\text{wf}} }}{{q_{\text{w}} }}$$

23 $$\text{MBT}_{\text{w}} = \frac{{W_{\text{p}} }}{{q_{\text{w}} }}$$

Equation (21) describe a linear relationship between RNP_w_ and MBT_w_, characterized by a unit slope on a log/log diagnostic plot. Once *c*_t_ is determined, the effective fracture volume (*V*_ef_) can be obtained from the RNP slope in the Cartesian coordinate. Therefore, *V*_ef_ in both FB_i_ and FB_s_ periods can be analyzed and compared.

If the flowing bottomhole pressure is higher than the bubble point pressure, the gas expulsion from the oil under downhole conditions is negligible. It indicates two-phase (oil and water) flow under the downhole conditions. Therefore, the multiphase flow model can be simplified as a two-phase flow and Eq. (19) can be simplified as

24 $$- q_{\text{w}} = V_{\text{ef}} \left[ {c_{\text{f}} + S_{\text{w}} c_{\text{w}} + S_{\text{o}} c_{\text{o}} } \right]\frac{\partial P}{{\partial t}}$$

As shown in Eq. (24), the most significant distinction between the two-phase model and the three-phase model is the absence of the gas term in the total compressibility.

## Model Application

### Reservoir and well completion information

To test the hypothesis presented in the methodology section, we analyze FB_i_ and FBs data from six parent wells completed in Niobrara and Codell formations in DJ Basin. The reservoir and well-completion data for these six wells are listed in Table 1. It includes total vertical depth (TVD), number of stages (*n*_s_), gross perforated interval (GPI), total injected proppant mass (*M*_prop_), total injected water volume (TIV), the shut-in period for initial flowback (*t*_sh_), the duration of production period (*t*_p_). TVD of the six wells varies from 2126 to 2202 m. These wells were fractured in 52–70 stages. *M*_prop_ is 4.69 to 4.76 × 10^6^ kg. Figure 1 schematically illustrates the operational events occurring during the life of these wells. *t*_sh_ represents the period from the completion date to the start date of initial flowback, ranging from 5 to 37 days. Compared to the other five wells, well 1 has the shortest *t*_sh_ of 5 days. *t*_p_ represents the interval from the end date of FB_i_ period to the start date of shut-in period before the FB_s_ period, ranging from 860 to 890 days.

**Table 1:** Reservoir and well-completion data

Well ID	TVD (m)	*n*_s_	GPI (m)	TIV (m^3^)	*M*_prop_ (10^6^ kg)	*t*_sh_ (d)	*t*_p_ (d)
Well 1	2171	69	3158	26,428	4.69	5	885
Well 2	2159	69	3158	26,284	4.69	22	869
Well 3	2202	52	3142	26,807	4.72	37	869
Well 4	2163	70	3167	26,332	4.76	26	874
Well 5	2126	69	3139	25,883	4.69	30	870
Well 6	2137	70	3167	26,009	4.76	20	875

### Bottomhole pressure and rate profiles

**Fig. 4:**
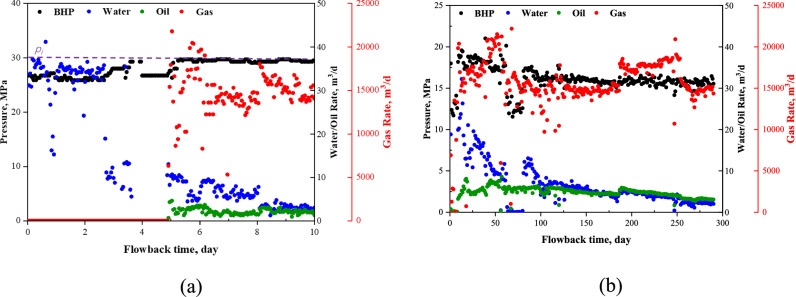
Bottomhole pressure and rate profiles of Well 5 during FB_i_ and FB_s_ periods. **a** Hourly measured initial flowback data; **b** Daily measured second flowback data. The duration of production period between the two flowback periods is 870 days

Figure 4a and b show the hourly measured FB_i_ data and the daily measured FB_s_ data of well 5 (the estimated bottom pressure, and flowrates of water, oil, and gas), respectively. The dashed line represents the estimated initial average reservoir pressure. There is a long period of single-phase water production before the oil and gas breakthrough during the FB_i_ period. The average bottomhole pressure in the FB_i_ period is about 20–30 MPa, while in the FB_s_ period it is about 10–20 MPa. The bottomhole pressure and rate profiles for the other five wells during FB_i_ and FB_s_ periods are shown in Appendix B.

**Fig. 5:**
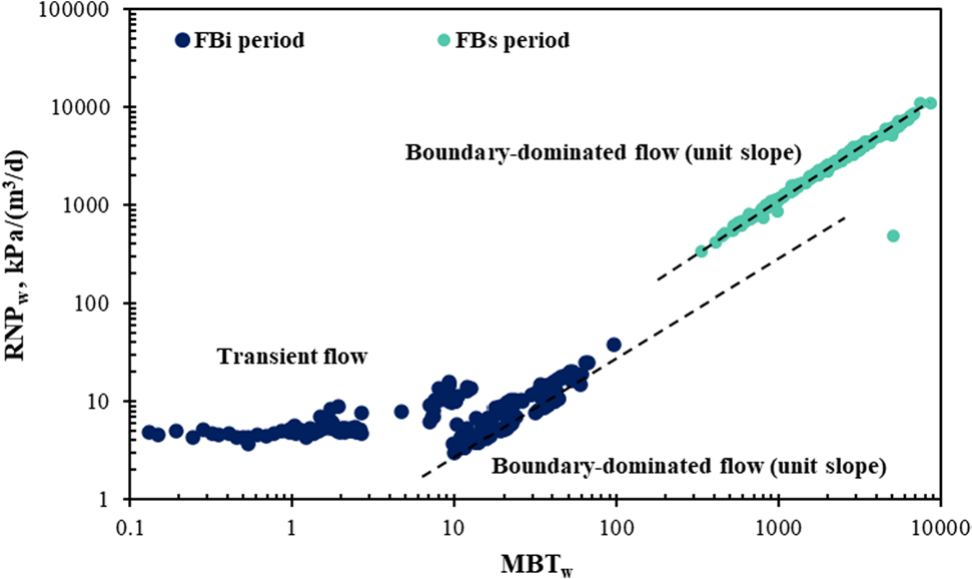
Preliminary analysis of the initial and secondary flowback periods

For the FB_i_ period, the long period of single-phase water production before the oil and gas breakthrough indicates that the fracture system is almost completely filled with fracturing water during the FB_i_ period. Second, the straight line with unit slope is observed in Fig. 5, suggesting the single-phase water is mainly from the fractures during the FB_i_. In other words, the water influx from the matrix into the fractures can be assumed to be negligible. During the FB_s_ period, the water is produced with hydrocarbon indicating that the fractures have multiphase fluids. The straight line with unit slope is also observed in the FB_s_ periods, indicating the water influx from matrix is negligible during FB_s_.

**Fig. 6:**
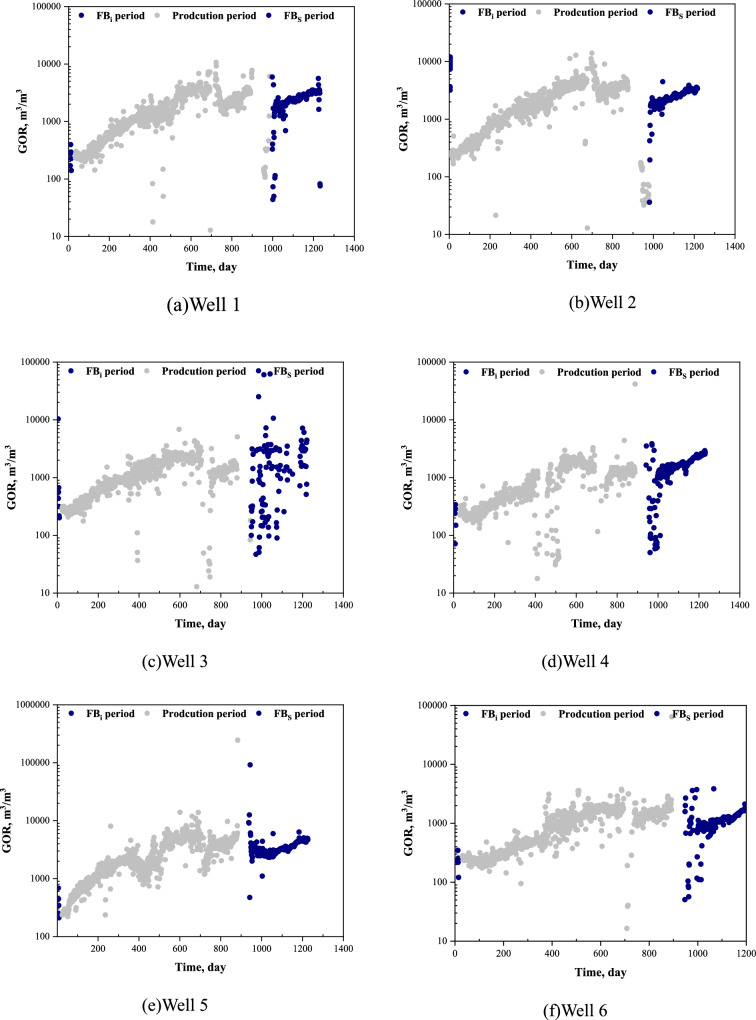
GOR profiles of six wells from FB_i_ to FB_s_ flowback periods

As shown in Figs. 6a–d and Fig. 6f, there is no significant increase in gas-oil ratio (GOR) for Well 1 ~ Well 4 and Well 6 from the start of FB_i_ period to the end of FB_s_ period. Although the bottomhole pressure decreases during the second flowback period, the insignificant changes in the GOR curve indicates that the bottomhole pressure is still higher than the bubble point pressure. In other words, the gas expulsion from the oil can be negligible under downhole conditions and the changes in fracture volume of these five wells can be analyzed using the simplified two-phase model. In contrast, Fig. 6e shows a significant increase (by more than an order of magnitude) in the GOR curve during the FBs period for Well 5, suggesting the presence of 3-phase flow (oil, gas and water) under the downhole conditions. Therefore, the multiphase flow model proposed in this paper needs to be adopted to accurately capture the changes in the fracture volume for well 5.

## Results and discussions

### Analyzing the FB_*i*_ and FB_s_ data

**Fig. 7:**
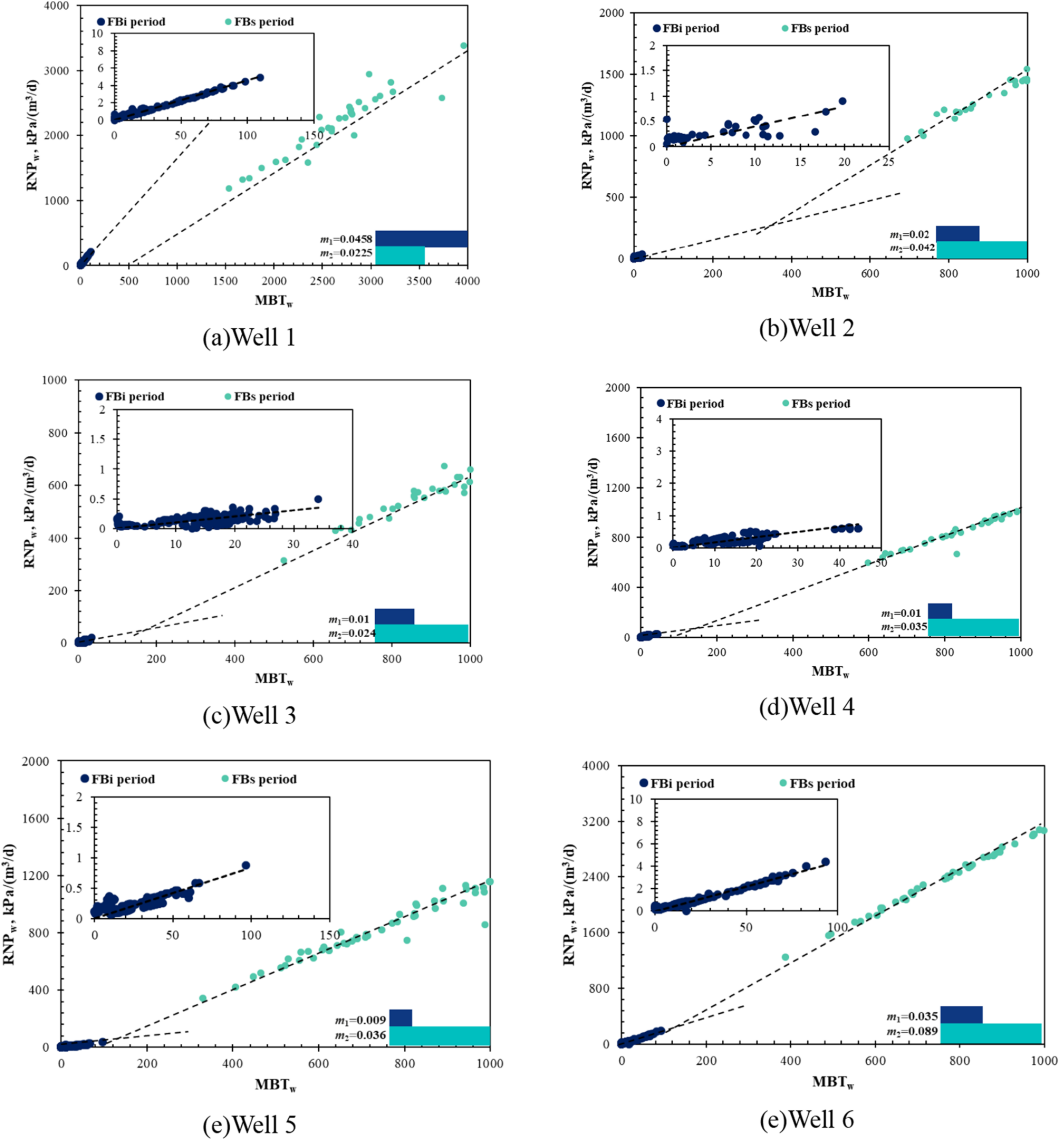
RNP vs MBT diagnostic plots of the six wells during the FBi and FBs periods

**Fig. 8:**
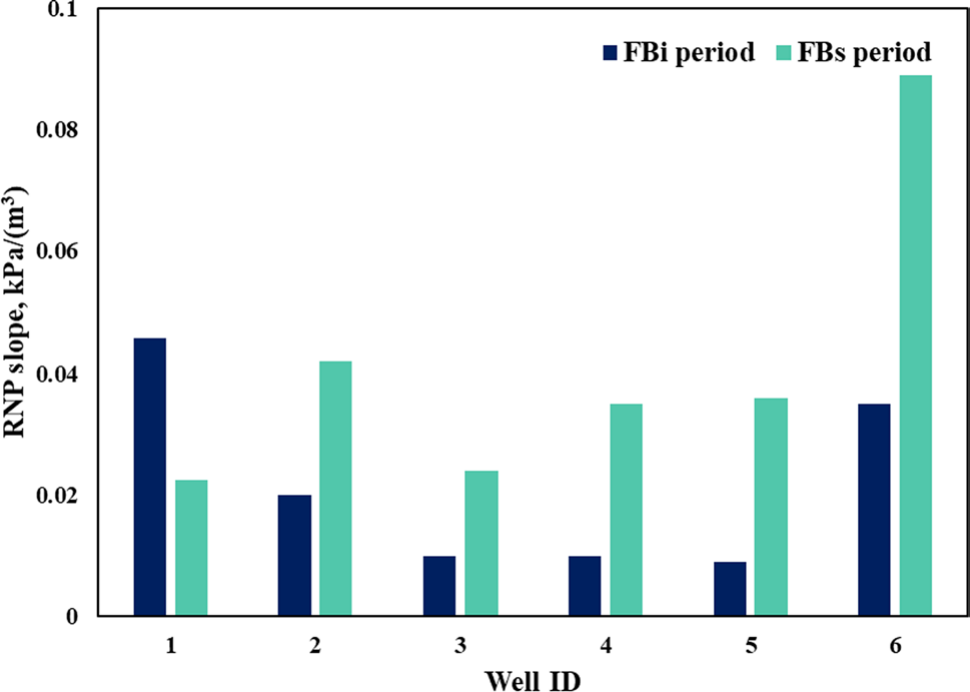
Comparing the RNP slope of the six wells during initial and second flowback periods

Figure 7 shows the RNP versus MBT diagnostic plots for the initial and second flowback periods of the studied parent wells. In order to quantitatively compare the flowback responses of the two periods, the RNP slopes for FB_i_ (*m*_1_) and for FB_s_ (*m*_2_) are compared in Fig. 8. In general, the RNP slope increases by approximately 2–5 times during the second flowback compared to the initial flowback, except for well 1. Interestingly, a straight line with unit slope is also observed in the RNP plot of the FB_s_ period where water is produced with hydrocarbons. This suggests negligible water influx from matrix and indicates that oil expansion serves as a drive mechanism for the pseudo-steady state flow of water, as described by Eq. (18). This also suggests that the fracture hit might have been avoided, as the closed-tank system of water is not interrupted by completing the child wells. Therefore, the increase in RNP slope with pressure depletion (5–10 MPa) from FB_i_ to FB_s_ period might be caused by the loss of effective fracture volume and the reduction in total compressibility due to partial fracture closure. The observed decrease in the RNP slope of well 1 may be attributed to an increase in total compressibility or the lower initial *V*_ef_ compared to the other wells,  as discussed later.

### Effective fracture volume estimation

**Fig. 9:**
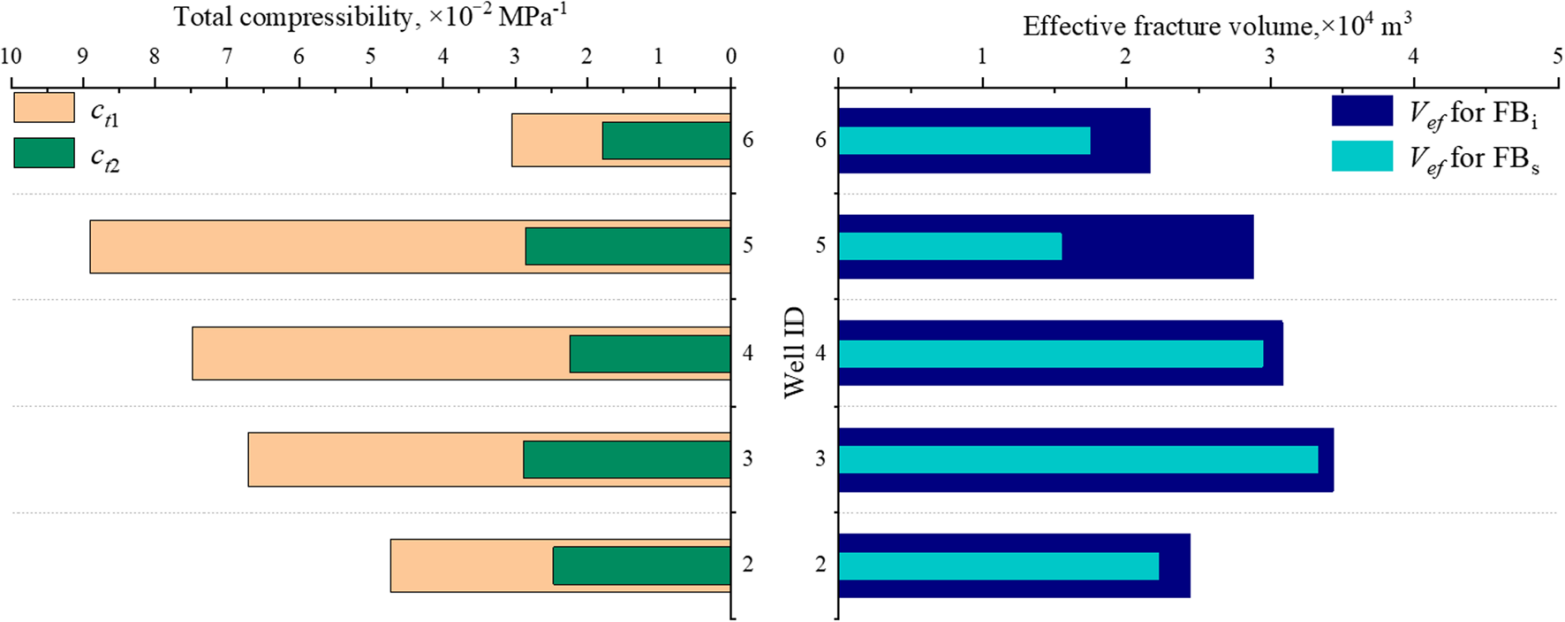
Estimations and variations of *V*_ef_ and *c*_t_ for the FB_i_ and FB_s_ periods. ${R}_{{c}_{\text{t}}}$ and ${R}_{{V}_{\text{ef}}}$ are defined by Eqs. (14) and (15)

Figure 9 shows the calculated *V*_ef_, ${R}_{{V}_{\text{ef}}}$, ${R}_{{c}_{\text{t}}}$ by analyzing the flowback data from the two flowback periods. ${R}_{{V}_{\text{ef}}}$ and ${R}_{{c}_{\text{t}}}$ are estimated using Eqs. (28) and (29). Compared to the FB_i_ period, *c*_t_ and *V*_ef_ for the five wells are decreased during the FB_s_ period. Although the total compressibility is decreased due to the pressure depletion from FB_i_ to FB_s_ periods, a loss of *V*_ef_ is still observed, with ${R}_{{V}_{\text{ef}}}$ values estimated at 3%–45%. The pressure profiles show that the bottomhole pressure is reduced by 5–10 MPa from the FB_i_ to the FB_s_ periods. Therefore, the high values of ${R}_{{V}_{\text{ef}}}$ may be attributed to fracture closure caused by pressure depletion between the two flowback periods (Wang et al. 2019). Moreover, the initial *V*_ef_ of well 1 is approximately 1.4 × 10^4^ m^3^, which is lower than the average *V*_ef_ for the other five wells. This may explain the relatively higher slope of RNP plot for well 1 during the FB_i_ period.

### Analyzing the production drive mechanisms

**Fig. 10:**
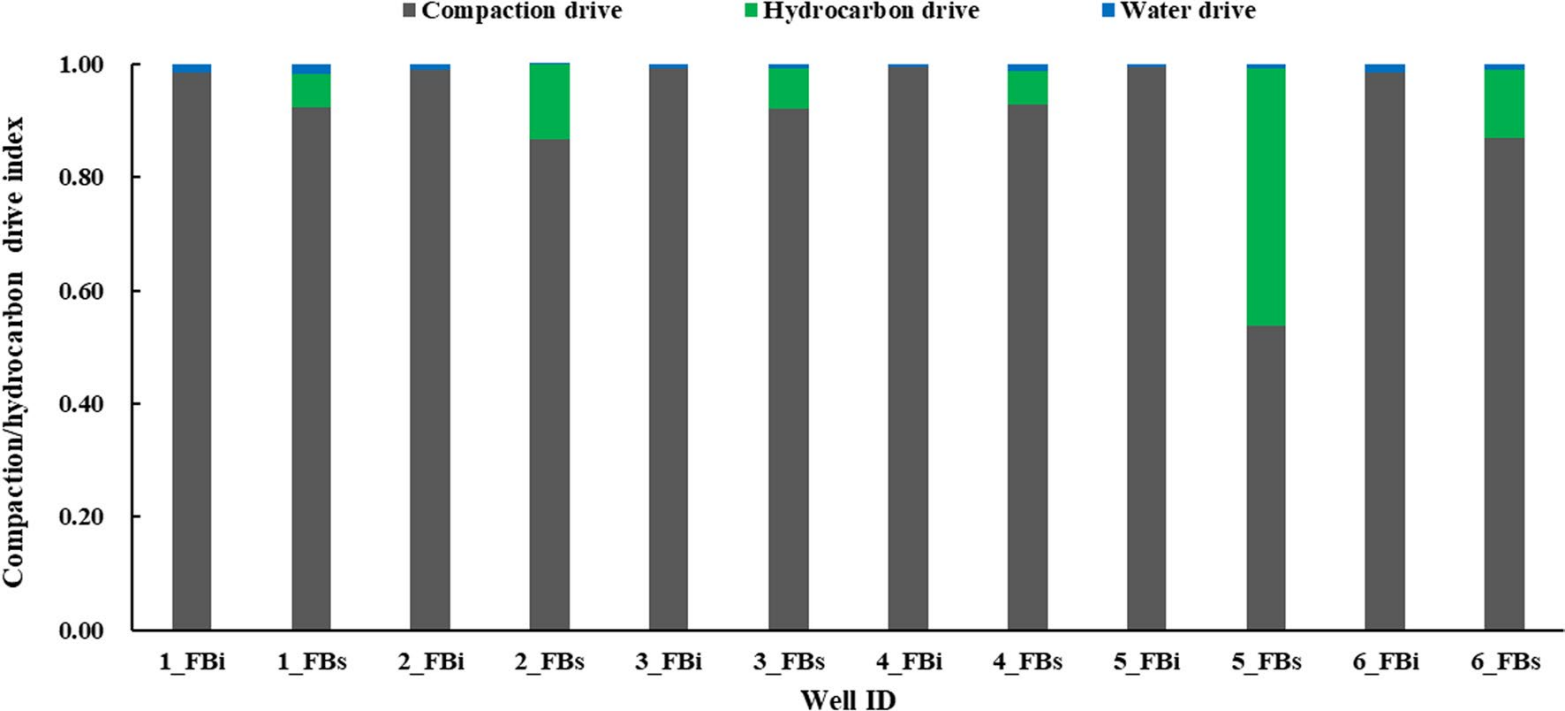
Variations of compaction-, water- and hydrocarbon-drive indices for the FB_i_ and FB_s_ periods

Based on the RNP slope, three drive indices are calculated for the initial and second flowback periods, as shown in Fig. 10. The compaction drive index (CDI), as the dominant mechanism, varies from 0.54 to 1 during the two flowback periods. However, compared to the initial flowback period, the CDI decreases by 16% on average for the five wells. In other words, the contribution of fracture closure as a driving mechanism is lower during the FB_s_ period compared with that during the FB_i_ period. The reduction in CDI is compensated by a 16% increase in EDI (EDI = HDI + WDI) during the FB_s_ period. For well 1, there is about 6% increase in HDI during the FB_s_ period. The greater hydrocarbon expansion might account for the decreased slope in the RNP versus MBT diagnostic plot during FB_s_. As a result, the overall increase in hydrocarbon compressibility for well 1 may explain the decrease in its RNP slope during the FBs period.

### Comparative analysis of two-phase and multiphase flow model for Well 5

To illustrate the difference in fracture volume analysis of FB_s_ between the multiphase model and the simplified two-phase model  considering the significant increase in GOR curve (by more than an order of magnitude), both models are applied to analyze the loss of fracture volume for well 5, as shown in Table 2.

**Table 2:** Comparative analysis of two-phase and multiphase flow model for Well 5

Parameters	Simplified two-phase model	Three-phase model
*S*_w_	0.635	0.635
*S*_o_	0.365	0.022
*S*_g_	/	0.343
*c*_t_	1.66 × 10^–4^	2.86 × 10^–4^
*V*_ef_ (m^3)^	26,603	15,440
CDI	0.92	0.54
HDI	0.06	0.46
WDI	0.01	0.01

As shown in Table 2, there is a significant deviation in the estimated fracture volume during the FBs period, which can be attributed to changes in total compressibility. In essence, the total compressibility depends on the variations in oil and gas saturation. Regarding the drive index, the increase in HDI compensates for the decrease in CDI. It is the result of the significant gas expulsion under downhole conditions. Therefore, the three-phase model is recommended for  analyzing fracture volume loss from FBi to FBs periods in the parent wells where the GOR increases significantly.

## Limitations and future work

This paper provides a workflow for evaluating the changes in fracture characteristics between initial and second flowback of parent wells. For the estimation of the effective fracture volume using the slope of RNP plots, the assumptions related to compressibility evaluation bring some uncertainties: (1) The initial free gas in fractures during FB_s_ is uncertain since the bubble-point pressure is not provided. (2) The fracture compressibility is obtained from the graphical method, where the approximation of fracture closure pressure and mineralization ratio both bring some uncertainties to the estimated fracture compressibilities. (3) The oil in fractures during the initial flowback period is neglected in this work, and the initial water saturation during the second flowback period is uncertain, both contributing to uncertainties in total compressibility. The relatively low number of parent wells makes it challenging to obtain accurate correlations between completion-design parameters and fracture volume loss. However, this work is a step forward in analyzing flowback data at different stages and comparing fracture characteristics. Future studies can focus on collecting a larger number of parent wells with reservoir, completion, and production data to estimate fracture compressibility and develop correlations between completion parameters and fracture volume loss.

## Summary and conclusions

The first and second flowback data from six parent wells were analyzed and compared to evaluate the changes in effective fracture volume during the production period. The RNP diagnostic plots of the six parent wells, completed in Niobrara and Codell formation, were constructed for both FBi and FBs periods to estimate the changes in effective fracture volume. Based on the analysis, the following conclusions were drawn:

1. The comparative analysis of the initial and second flowback data can be used to estimate the changes in effective fracture volume. This paper presents a  flowback model and workflow for estimating fracture volume changes, which includes flowback data preparations, bottomhole pressure estimations, RNP diagnostic analysis, average fracture volume estimation and  comparison of flowback drive mechanisms.
2. The RNP versus MBT diagnostic plots generally show an around 2–5 times increase in the slope during the second flowback period. The increase in RNP slope with pressure depletion (5–10MPa) from FB_i_ to FB_s_ period might be attributed to the loss of effective fracture volume and the reduction in total compressibility due to partial fracture closure.
3. The effective fracture volume during the two flowback periods can be quantitatively estimated using  the RNP slope and total compressibility. The percentage of effective fracture volume loss between the initial and second flowback periods is estimated at 3%–45%.
4. Compaction drive is identified as the dominant mechanism during both periods. The calculations show a 16% decrease in the compaction drive index during the second flowback period compared to  the initial period. The reduction in the compaction-drive index is compensated by an increase in the hydrocarbon-drive index during the second flowback period.

## Figures and Tables

**Fig. 11:**
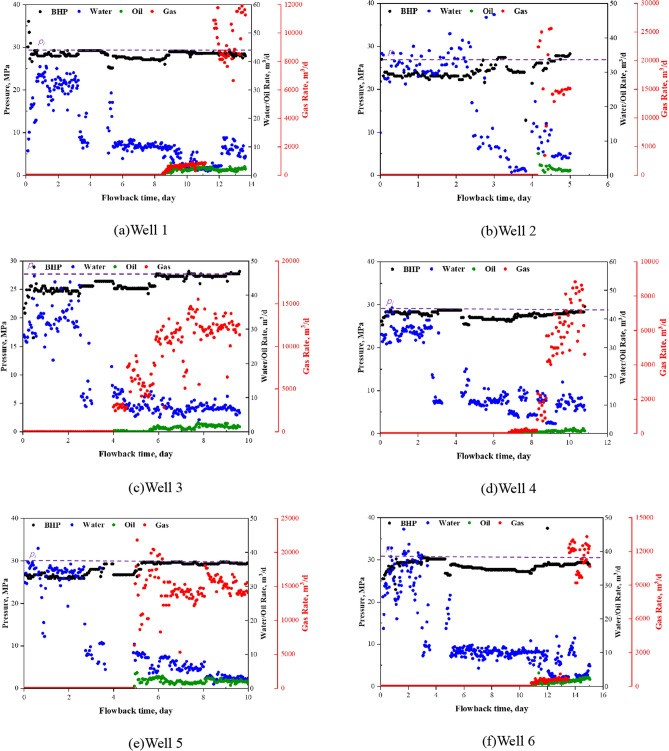
Bottomhole pressure and rate profiles for the remaining wells during FB_i_ period. **a** Well 1, **b** Well 2, **c** Well 3, **d** Well 4, **e** Well 5, **f** Well 6

**Fig. 12:**
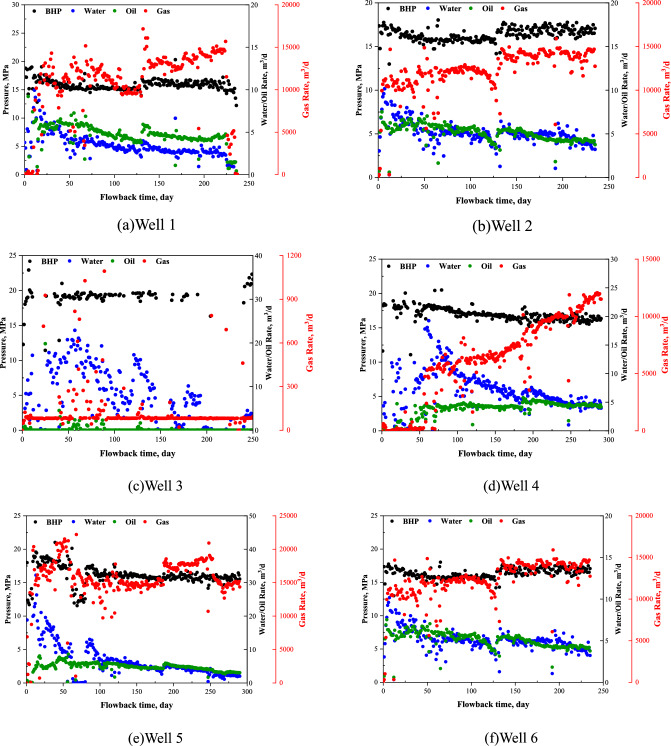
Bottomhole pressure and rate profiles during FB_s_ period. **a** Well 1, **b** Well 2, **c** Well 3, **d** Well 4, **e** Well 5, **f** Well 6

**Fig. 13:**
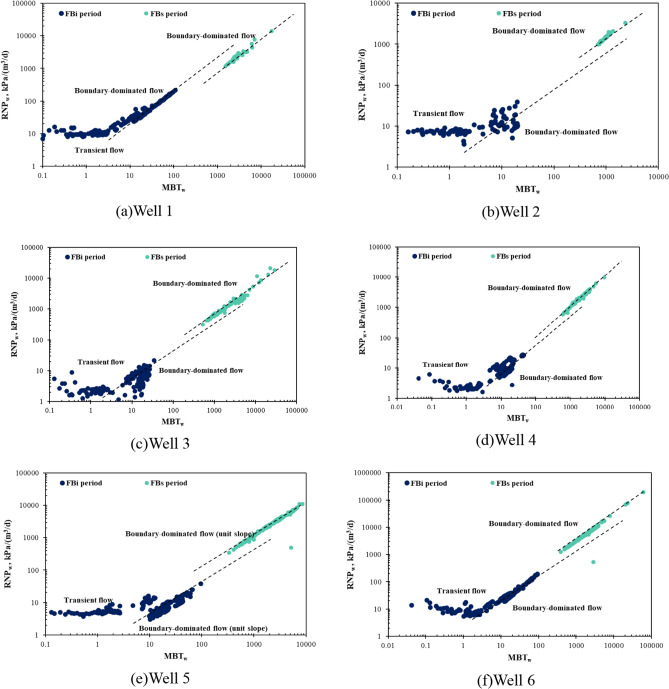
Comparative analysis of initial flowback and second flowback periods for other five wells. **a** Well 1, **b** Well 2, **c** Well, **d** Well 43, **e** Well 5, **f** Well 6

**Fig. 14:**
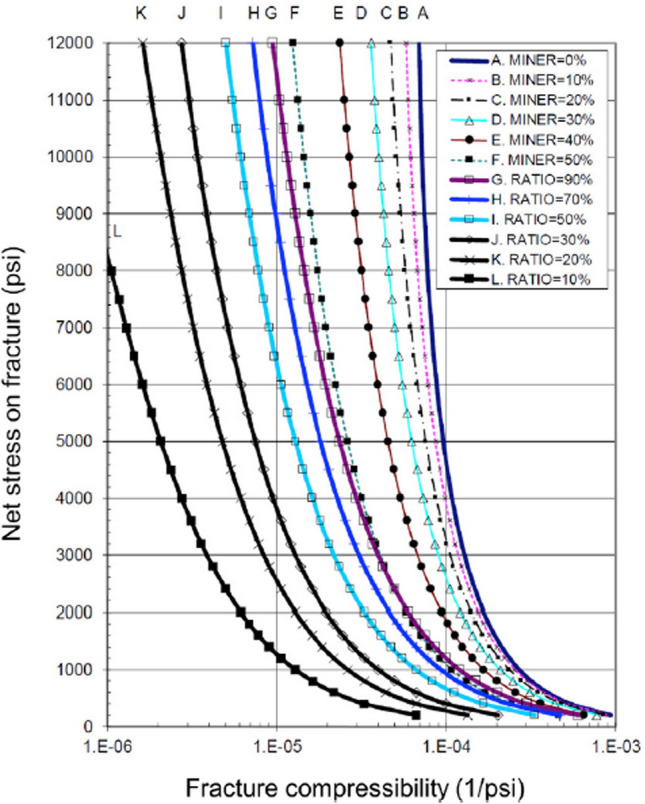
Type curves for estimating fracture compressibility proposed by Aguilera (1999)

## Data Availability

The data used to support the findings of this study are available from Hassan Dehghanpour upon request at dehghanpour@ualberta.ca.

## Appendix A Detailed derivations of flowing material equations for water in the fractures during the flowback periods

In this work, the fracture compressibility (*c*_f_) is defined as the changes in volume with respect to pressure whereas oil, gas, and water compressibility (*c*_o_, *c*_g_,* c*_w_) is defined as the changes in density with respect to pressure as:

A-1 $$c_{\text{o}} = \frac{1}{{\rho_{\text{o}} }}\frac{{\partial \rho_{\text{o}} }}{\partial P}$$

A-2 $$c_{\text{w}} = \frac{1}{{\rho_{\text{w}} }}\frac{{\partial \rho_{\text{w}} }}{\partial P}$$

A-3 $$c_{\text{f}} = \frac{1}{{V_{\text{ef}} }}\frac{{\partial V_{\text{ef}} }}{\partial P}$$

The water phase in the effective fracture volume is the focus of this work. Based on the material balance equation for water phase flow in fractures in Hossain et al (2019), we can obtain

A-4 $$\int\limits_{0}^{t} { - q_{\text{w}} \text{d}t = } \int\limits_{{p_{\text{i}} }}^{{p_{\text{wf}} }} {V_{\text{ef}} c_{\text{t}} \text{d}p}$$

Here, *c*_t_ is the total compressibility composed of the fracture, water, oil compressibility and water saturation.

A-5 $$c_{\text{t}} = c_{\text{pe}} { + }S_{\text{w}} c_{\text{w}} + S_{\text{o}} c_{\text{o}} + S_{\text{g}} c_{\text{g}}$$

From Eq. A-4, we can obtain

A-6 $$W_{\text{p}} = V_{\text{ef}} c_{\text{t}} (p_{\text{i}} - p_{\text{wf}} )$$

Both sides of Eq. A-6 are divided by the water rate at the same time, then Eq. A-18 can be rewritten as

A-7 $$\frac{{W_{\text{p}} }}{{q_{\text{w}} }} = \frac{{V_{\text{ef}} c_{\text{t}} (p_{\text{i}} - p_{\text{wf}} )}}{{q_{\text{w}} }}$$

Based on the definitions of RNP and MBT, then Eq. A-7 can be rewritten as

A-8 $$\text{RNP} = \frac{1}{{V_{\text{ef}} c_{\text{t}} }}\text{MBT}$$

## Appendix B The initial flowback and second flowback analysis

The bottomhole pressure and rate profiles for six wells during FB_i_ and FB_s_ flowback periods can be shown as Fig. ^11^ and Fig. ^12^.

The comparative analysis for six wells during the two flowback periods can be shown in Fig. ^13^.

## Appendix C Type curves for estimating fracture compressibility

See Figure 14.

## Abbreviations

*c*_p_: Proppant concentration, kg/m^3^
*M*_prop_: Total injected proppant mass, kg
*n*_s_: Number of stages, dimensionless
*p*_b_: Bubble point pressure, MPa
*p*_i_: Initial average pressure, MPa
*p*_wf_: Flowing bottomhole pressure, MPa
*q*_w_: Water rate, m^3^/d
*R*_Vef_: Loss of effective fracture volume, dimensionless
*t*_sh_: Shut-in time, day
*V*_ef_: Effective fracture volume, m^3^
*V*_ef__1_: Effective fracture volume of initial flowback period, m^3^
*V*_ef__2_: Effective fracture volume of second flowback period, m^3^
*W*_p_: Cumulative water production, m^3^
TVD: Total vertical depth, m
GPI: Gross perforated interval, m
TIV: Total injected water volume, m^3^
TIVF: Total injected water volume per foot, m^2^
CDI: Compaction-drive index, dimensionless
HDI: Hydrocarbon-drive index, dimensionless
WDI: Water-drive index, dimensionless
